# Supplementary material for: Profibrotic Inflammatory Cytokines and Growth Factors Are Predicted as the Key Targets of Uncaria gambir (Hunter) Roxb. in Keloids: An Epistatic and Molecular Simulation Approach
Source: Pharmaceuticals (Basel). 2024 May 21;17(6):662. doi: 10.3390/ph17060662 (PMC11206663; doi:10.3390/ph17060662)
Supplement: Supplementary file 1 [file pharmaceuticals-17-00662-s001.zip › pharmaceuticals-2947227-supplementary.pdf]

Table S1. Keloid-related target proteins score analysed by Cytoscape 3.9.1

No	Gene	Uniprotid	stringdbscore	ASPL	BC	CC	Clustering coefficient	Degree
1	AKT1	P31749	0.998	2.33	0.17	0.43	0.20	10
2	APC	P25054	0.996	3.30	0.00	0.30	0.00	1
3	APOL1	Q9BQ03	0.991	0.00	0.00	0.00	0.00	0
4	ASPN	Q9BXN1	0.992	0.00	0.00	0.00	0.00	0
5	ATP7A	Q04656	0.999	0.00	0.00	0.00	0.00	0
6	BMP1	Q99423	0.999	4.93	0.00	0.20	0.00	1
7	CAV1	Q03135	0.999	3.39	0.05	0.30	0.00	3
8	CCND1	P24385	0.988	2.72	0.02	0.37	0.33	4
9	CDK11B	Q9UP58	0.984	0.00	0.00	0.00	0.00	0
10	CDKN2A	P42771	0.987	3.17	0.00	0.32	0.00	2
11	COL1A1	Q15201	0.985	3.80	0.04	0.26	0.00	2
12	COL3A1	V9GZI1	0.985	4.78	0.00	0.21	0.00	1
13	COMP	P49747	0.99	0.00	0.00	0.00	0.00	0
14	CREBBP	Q92793	0.98	2.44	0.05	0.41	0.47	9
15	CTGF	P29279	0.985	2.94	0.03	0.34	0.17	4
16	CTHRC1	Q96CG8	0.985	0.00	0.00	0.00	0.00	0
17	CTNNB1	P35222	0.989	2.31	0.19	0.43	0.20	14
18	CXCL12	Q9H554	0.995	4.50	0.00	0.22	0.00	1
19	CXCR1	Q8N6T6	0.993	0.00	0.00	0.00	0.00	0
20	DCN	P07585	0.995	2.87	0.04	0.35	0.17	4
21	DDR1	Q5ST12	0.997	0.00	0.00	0.00	0.00	0
22	DDR2	Q16832	0.992	0.00	0.00	0.00	0.00	0
23	DPP4	P27487	0.993	3.87	0.01	0.26	0.00	2
24	EDN1	P05305	0.993	0.00	0.00	0.00	0.00	0
25	EGFR	Q9H2C9	0.997	2.57	0.13	0.39	0.00	6
26	EP300	Q09472	0.995	2.28	0.12	0.44	0.36	12
27	FAP	Q12884	0.999	0.00	0.00	0.00	0.00	0
28	FGF2	Q9UCS5	0.998	3.72	0.00	0.27	0.00	1
29	FLNA	P21333	0.982	4.37	0.00	0.23	0.00	1
30	FN1	H0Y4K8	0.992	3.00	0.14	0.33	0.05	7
31	FOXL2	P58012	0.999	0.00	0.00	0.00	0.00	0
32	FOXM1	Q08050	0.997	3.00	0.00	0.33	1.00	2
33	GDF9	O60383	0.997	0.00	0.00	0.00	0.00	0
34	HLA-DQA1	Q08AS3	0.998	1.00	0.00	1.00	0.00	1
35	HLA-DQB1	Q9MYH2	0.999	1.00	0.00	1.00	0.00	1
36	HLA-DRB1	Q5Y7B9	0.99	0.00	0.00	0.00	0.00	0
37	HMGB1	P09429	0.999	3.52	0.04	0.28	0.00	2
38	HSPA4	P34932	0.992	3.24	0.00	0.31	0.00	2

39	HSPA9	P38646	0.995	3.56	0.00	0.28	0.00	1
40	HSPB1	P04792	0.981	3.31	0.00	0.30	0.00	1
41	HTRA1	Q92743	0.996	0.00	0.00	0.00	0.00	0
42	IGF1R	P08069	0.999	2.83	0.06	0.35	0.00	3
43	IL10	P22301	0.999	3.02	0.00	0.33	0.67	3
44	IL17A	Q16552	0.999	3.20	0.00	0.31	0.67	3
45	IL6	P05231	0.983	2.85	0.01	0.35	0.33	4
46	KLF10	Q13118	0.999	0.00	0.00	0.00	0.00	0
47	LGALS1	P09382	0.999	0.00	0.00	0.00	0.00	0
48	LIN28B	Q6ZN17	0.999	0.00	0.00	0.00	0.00	0
49	MIF	P14174	0.997	0.00	0.00	0.00	0.00	0
50	MMP1	P03956	0.998	5.00	0.00	0.20	0.00	1
51	MMP13	P45452	0.999	0.00	0.00	0.00	0.00	0
52	MMP19	Q99542	0.999	0.00	0.00	0.00	0.00	0
53	MMP2	P08253	0.995	3.07	0.08	0.33	0.17	4
54	MT1A	P04731	0.996	0.00	0.00	0.00	0.00	0
55	MYH9	P35579	0.998	4.50	0.00	0.22	0.00	1
56	NEDD4	P46934	0.989	3.81	0.00	0.26	0.00	1
57	NLRC5	Q86WI3	0.991	0.00	0.00	0.00	0.00	0
58	NR3C1	P04150	0.988	2.81	0.02	0.36	0.50	4
59	NRG1	Q15491	0.984	3.56	0.00	0.28	0.00	1
60	PDGFA	P04085	0.992	0.00	0.00	0.00	0.00	0
61	PIK3CA	P42336	0.994	2.80	0.04	0.36	0.17	4
62	PIK3CB	P42338	0.99	3.28	0.00	0.31	1.00	2
63	PIK3CG	P48736	0.999	3.31	0.00	0.30	0.00	1
64	POSTN	Q3KPJ5	0.983	3.94	0.04	0.25	0.00	2
65	PTEN	P60484	0.989	2.83	0.05	0.35	0.30	5
66	PTGS1	Q5T7T8	0.999	0.00	0.00	0.00	0.00	0
67	PTN	P21246	0.998	0.00	0.00	0.00	0.00	0
68	RUNX2	Q13950	0.989	2.85	0.08	0.35	0.30	5
69	S100A12	P80511	0.999	0.00	0.00	0.00	0.00	0
70	S100A4	P26447	0.999	3.52	0.04	0.28	0.00	2
71	SERPINB2	P05120	0.998	0.00	0.00	0.00	0.00	0
72	SERPINB5	P36952	0.999	0.00	0.00	0.00	0.00	0
73	SERPINE1	P05121	0.985	0.00	0.00	0.00	0.00	0
74	SERPINH1	P50454	0.998	0.00	0.00	0.00	0.00	0
75	SFN	P31947	0.999	3.56	0.00	0.28	0.00	1
76	SFRP1	Q8N474	0.999	0.00	0.00	0.00	0.00	0
77	SKOR1	P84550	0.999	0.00	0.00	0.00	0.00	0
78	SMAD2	Q15796	0.993	2.46	0.04	0.41	0.71	7
79	SMAD3	P84022	0.993	2.28	0.11	0.44	0.44	11
80	SMAD4	Q13485	0.983	2.59	0.02	0.39	0.61	8
81	SMAD7	O15105	0.992	2.76	0.00	0.36	0.60	5
82	SPP1	P10451	0.996	3.98	0.00	0.25	0.00	1

83	STAT3	P40763	0.999	2.30	0.18	0.44	0.20	10
84	TAGLN	Q01995	0.999	0.00	0.00	0.00	0.00	0
85	TGFB1	P01137	0.998	2.56	0.11	0.39	0.21	8
86	TIMP1	P01033	0.99	4.02	0.04	0.25	0.00	2
87	TNC	P24821	0.988	0.00	0.00	0.00	0.00	0
88	TNFAIP6	P98066	0.999	0.00	0.00	0.00	0.00	0
89	TP53	Q8J016	0.999	2.57	0.26	0.39	0.14	12
90	TP63	Q9UP27	0.989	3.26	0.00	0.31	0.00	1
91	VDR	P11473	0.994	2.98	0.00	0.34	1.00	2
92	VEGFA	Q9H1W9	0.994	2.74	0.11	0.36	0.14	7
93	VIM	Q96ML2	0.994	0.00	0.00	0.00	0.00	0
94	WNT2	P09544	0.985	0.00	0.00	0.00	0.00	0
95	YAP1	P46937	0.996	2.85	0.03	0.35	0.17	4
96	ZC3H7B	Q9UK03	0.999	0.00	0.00	0.00	0.00	0

Table S2. Gambir bioactive compound target proteins

No	Bioactive Compound	Target protein
1	Quinic acid	HMGCR
2	Dimeric proanthocyanidin	VEGFA
	Dimeric proanthocyanidin	MMP13
	Dimeric proanthocyanidin	MMP12
	Dimeric proanthocyanidin	EGFR
3	Catechol	EGFR
	Catechol	MIF
	Catechol	IGF1R
4	Gambirflavan D1	VEGFA
5	Gambirflavan D2	VEGFA
	Gambirflavan D2	MMP13
	Gambirflavan D2	MMP12
	Gambirflavan D2	MMP14
6	Pyrocatechol	EGFR
	Pyrocatechol	MIF
	Pyrocatechol	IGF1R
7	Gambirine	MMP13
	Gambirine	EP300
	Gambirine	CCND1
	Gambirine	MMP16
8	Procyanidin B1	VEGFA
	Procyanidin B1	MMP13
	Procyanidin B1	MMP12
	Procyanidin B1	MMP14
	Procyanidin B1	NFKB
	Procyanidin B1	TIMP1

	Procyanidin B1	EGFR
	Procyanidin B1	MMP1
9	Procyanidin B2	VEGFA
	Procyanidin B2	MMP13
	Procyanidin B2	MMP12
	Procyanidin B2	MMP14
	Procyanidin B2	EGFR
	Procyanidin B2	MMP1
10	Gambiriin A1	VEGFA
	Gambiriin A1	MMP13
	Gambiriin A1	MMP12
	Gambiriin A1	MMP14
	Gambiriin A1	NFKB
	Gambiriin A1	PDGFA
	Gambiriin A1	EGFR
	Gambiriin A1	MMP1
11	Procyanidin B3	VEGFA
	Procyanidin B3	MMP13
	Procyanidin B3	MMP12
	Procyanidin B3	MMP14
	Procyanidin B3	NFKB
	Procyanidin B3	TGFB1
	Procyanidin B3	EGFR
	Procyanidin B3	MMP1
12	Procyanidin B4	VEGFA
	Procyanidin B4	MMP13
	Procyanidin B4	MMP12
	Procyanidin B4	MMP14
	Procyanidin B4	EGFR
	Procyanidin B4	MMP1
13	Gambiriin A2	VEGFA
	Gambiriin A2	MMP13
	Gambiriin A2	MMP12
	Gambiriin A2	MMP14
	Gambiriin A2	EGFR
	Gambiriin A2	TIMP1
	Gambiriin A2	MMP1
14	Gambiriin C	VEGFA
	Gambiriin C	MMP13
	Gambiriin C	MMP12
	Gambiriin C	MMP14
	Gambiriin C	EGFR
	Gambiriin C	MMP1
15	Tetrahydroalstonine	EGFR

	Tetrahydroalstonine	AKT1
	Tetrahydroalstonine	MMP13
	Tetrahydroalstonine	CCND1
16	4-methoxyphenol	MIF
	4-methoxyphenol	CCND1
	4-methoxyphenol	VEGFA
17	Phloroglucinol	EGFR
18	Protocatechuic acid	IGF1R
	Protocatechuic acid	EGFR
19	Isogambirine	EP300
	Isogambirine	MMP13
	Isogambirine	MMP10
	Isogambirine	MIF
	Isogambirine	CCND1
	Isogambirine	AKT1
20	Gambiriin A3	VEGFA
	Gambiriin A3	MMP13
	Gambiriin A3	MMP14
	Gambiriin A3	MMP12
	Gambiriin A3	IGF1R
	Gambiriin A3	EGFR

Table S3. Degree ranking of Gambir bioactive compound analysed by Cytoscape 3.9.1

No	Bioactive compound	BC	CC	Degree
1	Gambiriin A1	0.08	0.45	8
2	Procyanidin B1	0.06	0.45	8
3	Procyanidin B3	0.08	0.46	8
4	Gambiriin A2	0.04	0.45	7
5	Epicatechin gallate	0.07	0.41	6
6	Gambiriin A3	0.02	0.42	6
7	Gambiriin C	0.02	0.44	6
8	Isogambirine	0.09	0.4	6
9	Procyanidin B2	0.02	0.44	6
10	Procyanidin B4	0.02	0.44	6
11	Quercetin	0.02	0.4	5
12	4-allyl-2-methoxyphenol	0.03	0.37	4
13	Dimeric proanthocyanidin	0.01	0.4	4
14	Gambirflavan D2	0.01	0.38	4
15	Gambirine	0.08	0.36	4
16	Isoquercitrin	0.01	0.39	4
17	Kaempferol	0.01	0.38	4

18	Tetrahydroalstonine	0.03	0.4	4
19	4-methoxyphenol	0.02	0.35	3
20	Catechol	0.01	0.37	3
21	Hyperoside	0.01	0.38	3
22	Pyrocatechol	0.01	0.37	3
23	Dihydrocorynantheine	0	0.29	2
24	Protocatechuic acid	0	0.34	2
25	Ursolic acid	0.08	0.29	2
26	Gambirflavan D1	0	0.31	1
27	Phloroglucinol	0	0.33	1
28	Quinic acid	0	0.19	1
29	Uncariagambiriine	0	0.31	1
